# The VNTR of the *AS3MT* gene is associated with brain activations during a memory span task and their training-induced plasticity

**DOI:** 10.1017/S0033291720000720

**Published:** 2021-08

**Authors:** Wan Zhao, Qiumei Zhang, Xiongying Chen, Yang Li, Xiaohong Li, Boqi Du, Xiaoxiang Deng, Feng Ji, Chuanyue Wang, Yu-Tao Xiang, Qi Dong, Chuansheng Chen, Jun Li

**Affiliations:** 1 State Key Laboratory of Cognitive Neuroscience and Learning & IDG/McGovern Institute for Brain Research, Beijing Normal University, Beijing, P.R. China; 2 School of Public Health, Jining Medical University, 45# Jianshe South Road, Jining272013, Shandong Province, P.R. China; 3 The National Clinical Research Center for Mental Disorders & Beijing Key Laboratory of Mental Disorders & the Advanced Innovation Center for Human Brain Protection, Beijing Anding Hospital, School of Mental Health, Capital Medical University, Beijing 100088, China; 4 School of Mental Health, Jining Medical University, 45# Jianshe South Road, Jining272013, Shandong Province, P.R. China; 5 Faculty of Health Sciences, University of Macau, Avenida da Universidade, Taipa, Macau; 6 Department of Psychology and Social Behavior, University of California, Irvine, CA92697, USA

**Keywords:** AS3MT, brain activation, fMRI, plasticity, working memory training

## Abstract

**Background:**

The *Arsenic* (*+3 oxidation state*) *methyltransferase* (*AS3MT*) gene has been identified as a top risk gene for schizophrenia in several large-scale genome-wide association studies. A variable number tandem repeat (VNTR) of this gene is the most significant expression quantitative trait locus, but its role in brain activity *in vivo* is still unknown.

**Methods:**

We first performed a functional magnetic resonance imaging (fMRI) scan of 101 healthy subjects during a memory span task, trained all subjects on an adaptive memory span task for 1 month, and finally performed another fMRI scan after the training. After excluding subjects with excessive head movements for one or more scanning sessions, data from 93 subjects were included in the final analyses.

**Results:**

The VNTR was significantly associated with both baseline brain activation and training-induced changes in multiple regions including the prefrontal cortex and the anterior and posterior cingulate cortex. Additionally, it was associated with baseline brain activation in the striatum and the parietal cortex. All these results were corrected based on the family-wise error rate method across the whole brain at the peak level.

**Conclusions:**

This study sheds light on the role of *AS3MT* gene variants in neural plasticity related to memory span training.

## Introduction

The *Arsenic* (*+3 oxidation state*) *methyltransferase* (*AS3MT*) gene (10q24.32) encodes arsenite methyltransferase. This gene is expressed in human neurons and astrocytes (Burli et al., 2018; Duarte et al., 2016; Li et al., 2016), and it's level of expression in the human brain decreases gradually after birth and reaches a stable low level at ~20 years of age (Li et al., 2016). A recent study on multiple prefrontal cortex (PFC) RNA-seq samples identified a brain-specific isoform of this gene (*AS3MT* ^d2d3^) that lacks arsenite methyltransferase activity because it is missing 102 amino acids in the N-terminus (Li et al., 2016). This isoform is upregulated during human stem cell differentiation toward neuronal fates (Li et al., 2016). Individuals with certain neurodevelopmental disorders such as schizophrenia have more abundant *AS3MT* ^d2d3^ expression than controls (Li et al., 2016). Moreover, genome-wide association studies (GWASs) also supported the importance of *AS3MT* in the etiology of neurodevelopmental disorders such as schizophrenia. Some single-nucleotide polymorphisms (SNPs, rs7085104, rs11191914, and rs11191924) in the *AS3MT* promoter and nearby regions have been consistently identified as the top genome-wide significant risk variants for schizophrenia (Li et al., 2017; Ripke et al., 2013; Ripke et al., 2014; Yu et al., 2017).

To understand the biochemical functions of this gene, Li et al. (2016) did a fine-mapping analysis of the genetic markers across *AS3MT* and identified a variable number tandem repeat (VNTR) in the 5′ untranslated region of the first exon of *AS3MT* as one of the top expression quantitative trait loci (eQTL) for full-length *AS3MT* and *AS3MT* ^d2d3^. This VNTR is located 382 bp downstream of rs7085104 and is in strong linkage disequilibrium (LD) with all the reported GWAS risk SNPs within *AS3MT*. It is triallelic (two to four repeats) with each repeat composed of 36 bp. A study reported by Li et al. (2016), a larger number of repeats was associated with less methylation at a CpG island in exon 3 of *AS3MT* and greater expression of *AS3MT* ^d2d3^.

Taken together, *AS3MT* is clearly implicated in both normal neurodevelopment and neurodevelopmental disorders such as schizophrenia and its VNTR is the functional polymorphism. However, no research has explicated the neural basis of the link between this VNTR and the cognitive impairment associated with schizophrenia. The current study tested the contribution of the VNTR to brain activation during a working memory task and to neuroplasticity induced by a month-long working memory training. Because schizophrenia has been associated with reduced PFC activation during working memory (Minzenberg, Laird, Thelen, Carter, & Glahn, 2009) and disrupted neural plasticity (Forsyth & Lewis, 2017; Meyer-Lindenberg & Tost, 2014), we hypothesized that more repeats of this VNTR would be associated with lower PFC activation and plasticity.

## Methods

The protocol of this study was approved by the Institutional Review Board of the Institute of Cognitive Neuroscience and Learning at Beijing Normal University. All the subjects gave their written informed consent for this study.

### Subjects

Healthy undergraduate students (*N* = 101) were recruited from Beijing Normal University in China through an internet advertisement. They were interviewed by experienced psychiatrists to screen for potential mental disorders. All subjects received memory span training for 4 weeks (five 30- to 40-min sessions per week, 20 sessions in total) under the supervision of research assistants who were graduate students in psychology. They were administered a cognitive assessment and were scanned using functional magnetic resonance imaging (fMRI) both before and after the training.

### Training task

The training program was developed based on a visual-spatial span task (see online Supplementary Fig. S1A). Stimuli were green squares presented sequentially in a 5 × 5 gray grid (25 squares in total) on a computer screen. Each stimulus was presented for 500 ms with an interstimulus interval of 500 ms. After the presentation of the last stimulus, there was a 1000 ms intermission screen followed by an empty grid. Subjects were required to remember both the location and the order of all stimuli and to respond by repeating the sequence on an empty grid. The difficulty level was determined by the length of the sequence, which was automatically increased by 1 if subjects made five continuous correct responses on their current difficulty level. The training started with three stimuli. All subjects had to finish 80 trials for each session (lasting 30–40 min).

### fMRI task

The fMRI task included the memory condition and the baseline condition (see online Supplemental Fig. S1B). The stimuli were presented in cue-probe pairs. The memory condition used the same cue stimuli as those used for training (see above), except that the number of stimuli was set at 5. The probe stimulus was a green Arabic number within the empty grid. Subjects were asked to judge whether the Arabic number indicated the correct order of the cue stimuli. For the baseline condition, the cue stimuli were five red squares presented in the same order (top left corner→top right corner→bottom right corner→bottom left corner→center) across all trials. The probe stimuli for the baseline condition were the same as those for the memory condition except that the Arabic number was red. The fMRI task included 72 memory trials and 36 baseline trials (108 trials in total). Each trial started with a fixation cross for 500 ms, followed by five sequentially presented cue stimuli. Each cue stimulus was presented for 500 ms, with a 500 ms interstimulus interval. The total time for the presentation of cue stimuli was 4500 ms. After a 1000 ms intermission screen, probe stimuli were presented for 2000 ms, during which subjects made their responses using a fiber-optic response box. Subjects pressed the left button if the Arabic number at a given location correctly reflected the sequence number of the cue stimuli and pressed the right button if the Arabic number was incorrect.

### fMRI data acquisition

All imaging data were acquired at the Brain Imaging Center of Beijing Normal University. All subjects were scanned both before and after training. Subjects were scanned on a Siemens TIM Trio 3T scanner (Siemens, Erlangen, Germany) with their head snugly fixed with straps and foam pads to restrict head movement. Functional images during the performance of the spatial span task as described above were collected axially using the following echo-planar imaging sequence: repetition time (TR) = 2000 ms; echo time (TE) = 30 ms; flip angle (FA) = 90^o^; field of view (FOV) = 200 × 200 mm^2^; matrix size = 64 × 64; axial slices = 31; 4.0 mm slice thickness without gap (i.e. interleaved scan); and voxel size = 3.1 × 3.1 × 4.0 mm^3^.

### fMRI data preprocessing

Data preprocessing was implemented using Statistical Parametric Mapping software (SPM version 12.0, Wellcome Department of Cognitive Neurology, London, UK). Preprocessing included realignment (correcting for head movement; any subject with more than 2 mm translation or 2° rotation was excluded), normalization (to the Montreal Neurological Institute space), resampling (to a voxel size of 3 × 3 × 3 mm^3^), and spatial smoothing (with 8 mm full-width at half maximum of the Gaussian smoothing kernel). We focused our analysis on the cue phase (from the start of the cue stimuli to the start of the probe stimuli), which covered cognitive processing of both encoding and maintenance during working memory. In the first-level (within-subjects) analysis, we first used task condition (memory *v*. baseline) as a predictor to produce brain activation images for each subject at each time point. The resulting images at pretest were entered into the second-level (between-subjects) data analysis to examine the association between the VNTR and pretest brain activation. We next analyzed changes in brain activation from pre- to post-training scans. In these analyses, a high-pass filter at 128 s was used to remove noise associated with low-frequency confounds. The resulting images were entered into the second-level data analysis on the association between the VNTR and brain activation plasticity.

### Genotyping

Saliva samples were collected using an Oragene-DISCOVER Kit (DNA Genotek, Canada) from all subjects after they finished their pretest. Genomic DNA was extracted using the standard method supplied by the same kit. The polymerase chain reaction (PCR) for the triallelic VNTR was performed using a 9700 thermocycler (Applied Biosystems, USA) in 10 *μ*l amplification mixture containing 0.5 mm of each primer (forward: 5′-CGGAGCAAGCCTGCCAG-3′ and backward: 5′-TGAGGGGACGACAAAGG-3′), 1× AmpliTaq Gold™ 360 Master Mix (Applied Biosystems, USA), and 30 ng template DNA. After an initial 5 min at 96 °C, the PCR proceeded with 36 cycles of 96 °C for 30 s, 51 °C for 30 s, and 72 °C for 30 s. After a final 10 min at 72 °C, the PCR was terminated at 4 °C. The PCR products for two, three, and four repeats were 240 bp, 276 bp, and 312 bp, respectively. They were separated by electrophoresis in 3% agarose gels. Each sample was genotyped twice. Genotypes were read by two researchers. Of the total sample, we observed only three samples with the four-repeat allele. We excluded them from further data analysis due to the small sample size.

### Data analysis

One-way ANOVA and χ^2^ test in Statistical Product and Service Solutions software (SPSS version 22.0, SPSS Inc., Chicago, Illinois, USA) were used to compare genotypic differences in terms of demographic variables and behavioral data.

In the SPM 12.0 analyses of the fMRI data, we first conducted regression analyses to test the association between the VNTR and brain activation. Genotype (2R/2R *v*. 2R/3R *v.* 3R/3R) was entered as an independent factor, and brain activation (working memory condition minus baseline condition) at pretest as the dependent factor. The significance level was set at *p* < 0.05 after whole-brain family-wise error (FWE) correction at the peak-level.

We also conducted regression analyses to test the genotypic effect on brain activation plasticity. In this analysis, genotype (2R/2R *v.* 2R/3R *v.* 3R/3R) was entered as an independent factor, and training-induced activation changes (posttest minus pretest) as the dependent factor. The significance level was also set at *p* < 0.05 after whole-brain FWE correction at the peak-level. For the clusters showing significant genotypic effects, we extracted their activation values at both pretest and posttest to do *post hoc* repeated measures ANOVA in SPSS 22.0.

## Results

No deviation from the Hardy–Weinberg equilibrium was found (*p* > 0.05). As described above, three subjects carrying four repeats allele were excluded. Five additional subjects were excluded due to their excessive head motion (>2° or 2 mm) during either pre- or post-training fMRI scans, yielding a final sample of 93 subjects. In terms of genotype, there were 24 2R/2R, 47 2R/3R, and 22 3R/3R individuals. The three genotype groups were comparable on all demographic factors (all *p*_s_ > 0.05) (see Table 1). Behavioral performance on the fMRI task at both pretest and posttest was comparable between genotypes (all *p*_s_ > 0.05) (see Table 1).

**Table 1.** Demographic variables and behavioral performance across the three VNTR groups

	Mean ± s.d.			*F* or χ^2^	*p*
	2R/2R	2R/3R	3R/3R		
Number of subjects	22	47	24		
Gender (male/female)	5/17	11/36	4/20	0.45	0.797
Age (years)	21.05 ± 2.17	22.23 ± 2.335	22.12 ± 2.53	2.02	0.138
Education (years)	15.00 ± 1.63	15.79 ± 1.94	15.63 ± 2.28	1.21	0.302
IQ^a^	131.05 ± 4.73	129.85 ± 5.81	129.17 ± 6.57	0.62	0.540
fMRI task performance at pretest^b^	0.91 ± 0.07	0.89 ± 0.08	0.90 ± 0.06	0.05	0.948
fMRI task performance at posttest^b^	0.96 ± 0.05	0.92 ± 0.07	0.94 ± 0.05	3.06	0.052

a Full scale IQ, as measured by Wechsler Adult Intelligence Scale.

b Using age, gender, and education as covariates.

**Fig. 1.**
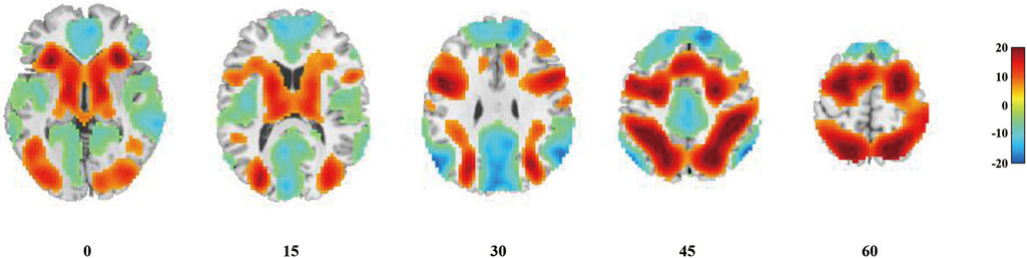
Significant genotypic effects on baseline brain activation based on whole-brain analysis. Individuals with the three-repeat allele showed significantly lower activation within the regions belonging to the ECN (i.e. bilateral PFC and bilateral parietal cortex, in warm colors) and some subcortical regions that have been reported to be activated during learning and working memory (i.e. bilateral striatum, in warm colors). Additionally, individuals with the same allele showed lower deactivation within regions belonging to the DMN (i.e. the ACC and the PCC, in cool colors).

The VNTR was significantly associated with baseline brain activation in several brain regions that are known to be important for working memory (see Fig. 1). Specifically, the three-repeat allele was associated with lower baseline activation within the bilateral PFC, parietal cortex, and striatum, and it was associated with less baseline deactivation within both the anterior cingulate cortex (ACC) and the posterior cingulate cortex (PCC) (see Fig. 1, peak-level FWE corrected *p_s_* < 0.001).

**Fig. 2.**
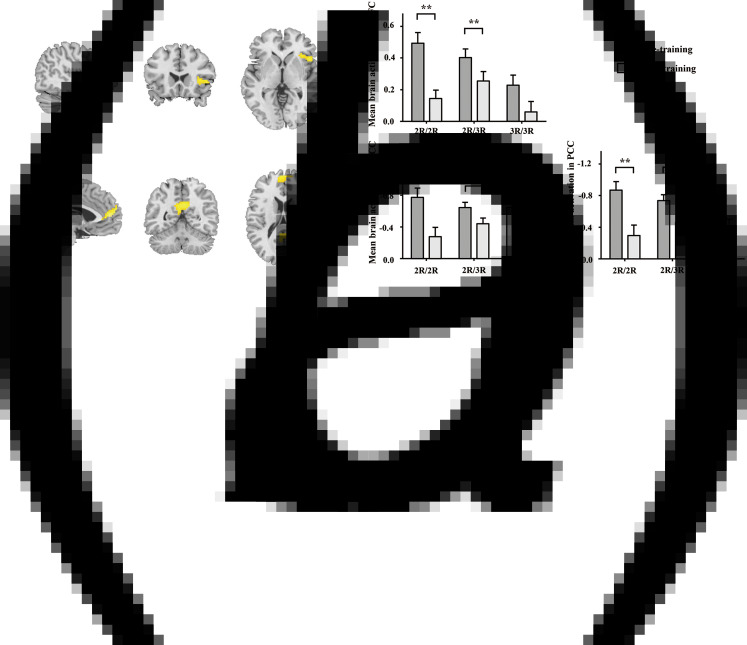
Significant genotypic effects on training-related neural plasticity. Panel (*a*) shows that the three-repeat allele, which has been associated with the upregulated expression of *AS3MT* ^d2d3^, was significantly associated with less activation reduction within the right ventrolateral PFC (62 voxels, *x* = 48, *y* = 18, *z* = 3, peak-level FWE corrected *p* < 0.001). Panel (*b*) shows that the three-repeat allele was associated with less deactivation reduction within both the ACC (137 voxels, *x* = −3, *y* = 48, *z* = 9, peak-level FWE corrected *p* < 0.001) and the PCC (292 voxels, *x* = −3, *y* = −45, *z* = 30, peak-level FWE corrected *p* < 0.001), both of which were important components of the DMN.

In terms of training-induced changes, the three-repeat allele was associated with smaller activation changes after the training in three regions, including the right ventrolateral PFC (62 voxels, *x* = 48, *y* = 18, *z* = 3, peak-level FWE corrected *p* < 0.001, see Fig. 2*a*), ACC (137 voxels, *x* = −3, *y* = 48, *z* = 9, peak-level FWE corrected *p* < 0.001, see Fig. 2*b*) and PCC (292 voxels, *x* = −3, *y* = −45, *z* = 30, peak-level FWE corrected *p* < 0.001, see Fig. 2*b*). These three regions overlapped with those that showed VNTR-related baseline brain activation mentioned above. For the right ventrolateral PFC significant cluster, 41 out of the 62 voxels' baseline activations were significantly associated with the VNTR (peak-level FWE corrected *p* < 0.001, see online Supplementary Fig. S2). For both the ACC and PCC significant clusters, all voxels' baseline activations were significantly associated with the VNTR (peak-level FWE corrected *p_s_* < 0.001).

Activation values at both pretest and posttest for the three regions were then extracted. The activation level at the right ventrolateral PFC was reduced after the training (*F* = 25.29, *p* < 0.001). The 2R/2R group and the 2R/3R group showed significant PFC activation reduction after the training (for the 2R/2R group, *F* = 18.02, *p* < 0.001; for the 2R/3R group, *F* = 10.78, *p* = 0.002), but the 3R/3R genotype group showed a similar but not significant trend (*F* = 2.64, *p* = 0.118). The deactivation level at the ACC (*F* = 30.69, *p* < 0.001) and PCC (*F* = 32.54, *p* < 0.001) was also reduced after the training. For the ACC, all three genotype groups showed significant deactivation reduction (for the 2R/2R, *F* = 13.61, *p* = 0.001; for the 2R/3R, *F* = 10.69, *p* = 0.002; for the 3R/3R, *F* = 7.37, *p* = 0.012), although the change in the 2R/2R genotype was the most significant. For the PCC, the two genotypes carrying the two-repeat allele showed significant deactivation reduction (for the 2R/2R, *F* = 14.60, *p* = 0.001; for the 2R/3R, *F* = 19.20, *p* < 0.001), but the 3R/3R genotype group showed only a similar but not significant trend (*F* = 3.19, *p* = 0.087).

## Discussion

The current fMRI study tested the contribution of a schizophrenia risk variant within the *AS3MT* gene to both baseline brain activation and training-related brain activation plasticity. We focused on a VNTR that is near, and in very strong LD with, *AS3MT* SNPs (e.g. rs7085104, rs11191914, and rs11191424) that have genome-wide significant associations with schizophrenia. This VNTR has been reported to be the most significant eQTL for *AS3MT*^d2d3^, a schizophrenia-specific isoform involved in neural development (Li et al., 2016). We found that the VNTR was significantly associated with both baseline brain activation and brain activation plasticity at three closely related regions, including the right ventrolateral PFC, the ACC, and the PCC.

Consistent with our hypothesis, we found that the three-repeat allele of the VNTR was associated with PFC hypoactivation at the baseline condition. This result is consistent with two recent meta-analyses that have suggested much lower PFC activation in schizophrenia patients and/or their healthy relatives than in healthy controls during working memory (Minzenberg et al., 2009; Zhang, Picchioni, Allen, & Toulopoulou, 2016). A study that compared brain activation during working memory encoding and maintenance processes between schizophrenia patients and healthy controls also confirmed this result (Anticevic, Repovs, & Barch, 2011). Based on such findings, the current study focused on the encoding and maintenance processes of working memory and found that individuals with the three-repeat allele failed to recruit PFC to the same extent as those with the two-repeat allele.

Moreover, we found that the three-repeat allele was associated with less PFC activation reduction induced by the training. This finding, together with altered expression of *AS3MT*^d2d3^ during neural development, suggested that this gene plays an important role in neuroplasticity. As reported by several randomized controlled studies, working memory training decreases brain activation at the PFC (Brehmer et al., 2011; Salmi, Nyberg, & Laine, 2018). Our current results also support that conclusion. Less decreased activation of the PFC after training indicated less plasticity in this region. The association between the three-repeat allele and less PFC plasticity was then consistent with the neuroplasticity hypothesis of schizophrenia (Forsyth & Lewis, 2017), which states that schizophrenia patients not only exhibit a developmental delay in some brain regions including the PFC (Harris et al., 2007; Thermenos et al., 2013) but also exhibit decreased brain reactivity to changing environmental demands (Li et al., 2015; Meyer-Lindenberg & Tost, 2014). Because the memory span training led to reduced PFC activation, it seems that training improved PFC efficiency rather than mobilizing more PFC resources. In accordance with this speculation, we might infer that individuals with the three-repeat allele might have had insufficient PFC involvement at the baseline condition and benefit less from the training compared to individuals with the two-repeat allele. This may be a reason for the observed association between the three-repeat allele and less PFC plasticity. In sum, possible mechanisms for the contributions of this VNTR to schizophrenia may involve less recruitment of the PFC and also impaired PFC plasticity for individuals with the risk variant.

In addition to the PFC, our results also highlighted the importance of the ACC and PCC for the role of *AS3MT* in brain function. Unlike the PFC, which is an important component within the executive control network (ECN) and is activated during working memory, both the ACC and PCC belong to the default mode network (DMN) and are deactivated (i.e. suppressed) when performing a working memory task. Deactivation of the DMN suppresses mind wandering and other kinds of spontaneous thought, which is helpful for the brain to reallocate its limited resource to the ECN (Fox, Spreng, Ellamil, Andrews-Hanna, & Christoff, 2015; *Huijbers* et al., 2012) to perform the ongoing working memory task. In addition to reduced activation of the PFC, reduced deactivation of the DMN has also been associated with worse working memory encoding and maintenance processes in patients with schizophrenia (Anticevic et al., 2011). Consistently, we found that the three-repeat allele was associated with less baseline DMN deactivation in addition to less baseline PFC activation. In fact, our previous studies about another schizophrenia risk gene polymorphism (*MIR137* rs1625579) also found an association between the risk variant and less deactivation of the DMN (Zhang et al., 2018). For the role of the VNTR in plasticity of both the ACC and PCC, it is again the three-repeat allele that was associated with less baseline recruitment (i.e. less deactivated) further showed less deactivation reduction. Previous studies have suggested that the deactivation within the DMN was reduced in response to lower cognitive demands (Mckiernan, Kaufman, Kucera-Thompson, & Binder, 2003; Park, Polk, Hebrank, & Jenkins, 2010). The working memory training reduced the difficulty of the same fMRI task, which in turn reduced the deactivation of the DMN. Accordingly, less reduction of the DMN deactivation indicated less DMN plasticity. Therefore, the association between the three-repeat allele and less deactivation reduction after the training within the DMN suggested worse DMN plasticity for individuals with the risk allele. In sum, in addition to the right ventrolateral PFC, less recruitment and impaired plasticity within both the ACC and PCC also contributed much to the predisposition of the *AS3MT* risk variant to schizophrenia.

Three other issues need to be mentioned. First, although the VNTR was associated with baseline brain activation in the parietal cortex and the striatum, it did not show any association with the plasticity of these regions. Considering that the PFC plays a different role in working memory (i.e. active maintenance of working memory) compared with both the parietal cortex (working memory capacity) and the striatum (working memory updating), it seems that the VNTR may only affect the plasticity of a specific working memory component. Second, our finding about the association between the three-repeat allele and impaired brain activation plasticity may partly explain why individuals with genetic susceptibility to schizophrenia are prone to be triggered by stressful events (Beaton & Simon, 2011; Gomes & Grace, 2017; Howes, McCutcheon, Owen, & Murray, 2017). The working memory training task could partly mimic the high-stress pathogenic environment of schizophrenia. As is known, individuals with high vulnerability for schizophrenia are prone to symptoms under high stress (Beaton & Simon, 2011; Gomes & Grace, 2017; Howes et al., 2017) because of their inability to dynamically adapt their brain to the changing environmental demands (Li et al., 2015; Meyer-Lindenberg & Tost, 2014). The training involved in the current study was of an adaptive style, which was likely to place trainees in a high-stress environment by keeping the working memory task at a difficulty level that is close to their capacity. This may be an additional advantage of using training-related plastic changes as an index of brain functions of patients with schizophrenia. Finally, the current study included only healthy participants because of the high demand of the training, and thus any implications of the results for our understanding of schizophrenia are tentative. Future research should adapt our methods so they can be used with patients with schizophrenia.

In conclusion, this study suggested that the schizophrenia risk variant of the *AS3MT* gene was associated with not only less brain function but also less brain functional plasticity, especially at three closely related regions (i.e. the right ventrolateral PFC, the ACC, and the PCC). These results deepened our understanding of the role of *AS3MT* in neural plasticity related to memory span training.

## Supplementary material

For supplementary material accompanying this paper visit https://doi.org/10.1017/S0033291720000720. click here to view supplementary material
