# Supplementary material for: The VNTR of the AS3MT gene is associated with brain activations during a memory span task and their training-induced plasticity
Source: Psychol Med. 2020 Apr 20;51(11):1927–32. doi: 10.1017/S0033291720000720 (PMC8381288; doi:10.1017/S0033291720000720)
Supplement: Supplementary file 1 [file S0033291720000720sup.zip › S0033291720000720sup003.docx]

**SUPPLEMENT** **FIGURE LEGEND**

Figure S1. The training version (Panel A) and the fMRI version (Panel B) of the memory span task. During training, subjects were required to remember both the location and the order of all stimuli and to tap the squares in the empty grid to indicate their locations in the order they were presented. The number of stimuli was kept at 3 for the control group. For the adaptive training group, the number of stimuli started with 3 but was automatically increased by 1 if the subject showed 5 continuous correct responses on the current difficulty level. For the fMRI version, subjects were required to judge whether the Arabic number indicated the correct order of the cue stimuli that were presented. Stimuli were green in the memory condition but were red in the baseline condition.

Figure S2. The VNTR-related training-induced activation changes overlapped with the VNTR-related baseline activation in the PFC. The orange region was the region with overlapping baseline activation and training-induced activation changes (41 voxels), and the yellow region and the orange region together showed the training-induced activation changes (62 voxels).
