# Supplementary figures and images for: The VNTR of the AS3MT gene is associated with brain activations during a memory span task and their training-induced plasticity
Source: Psychol Med. 2020 Apr 20;51(11):1927–32. doi: 10.1017/S0033291720000720 (PMC8381288; doi:10.1017/S0033291720000720)

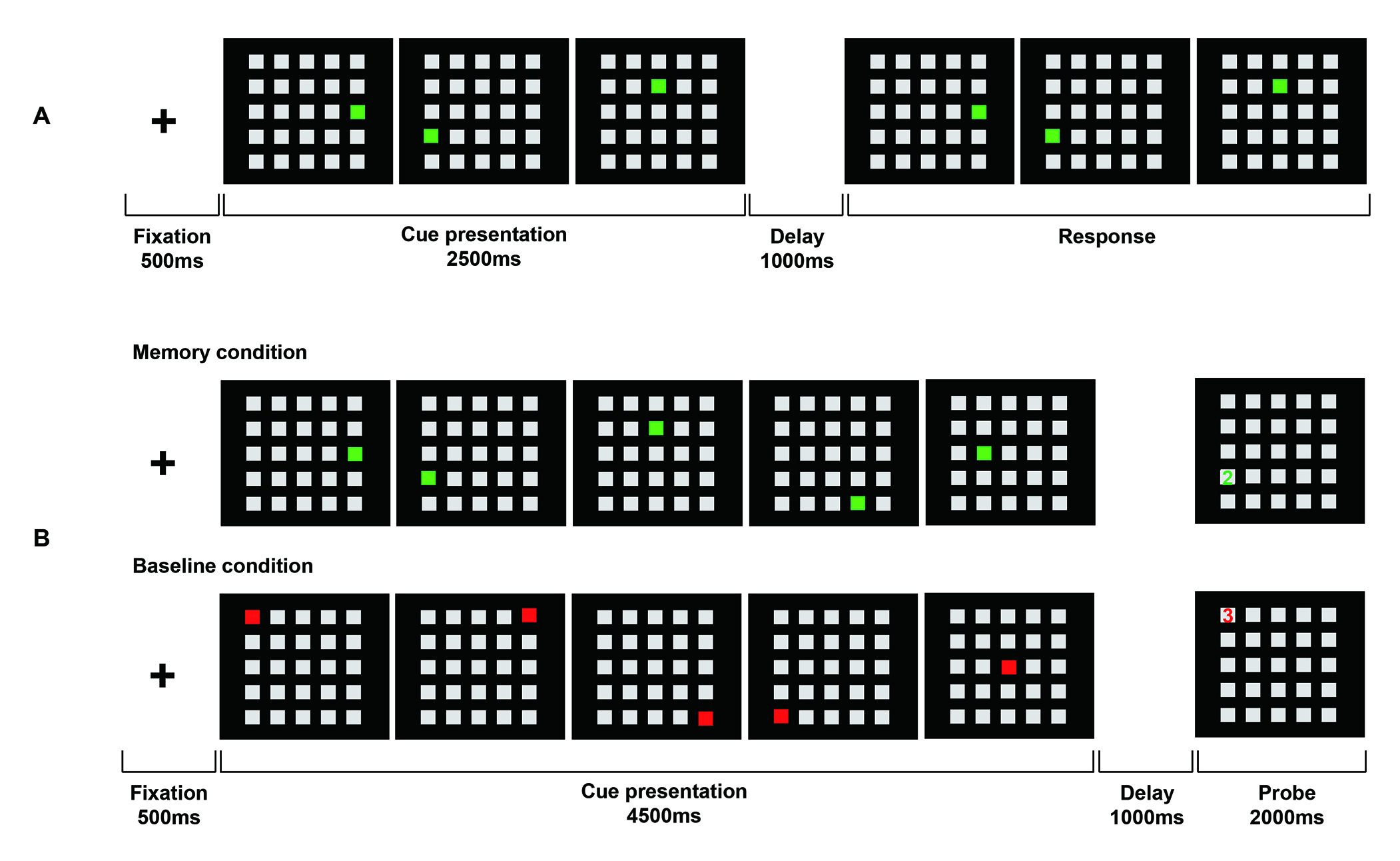

Supplement: Supplementary file 1 [file S0033291720000720sup.zip › S0033291720000720sup001.tif]

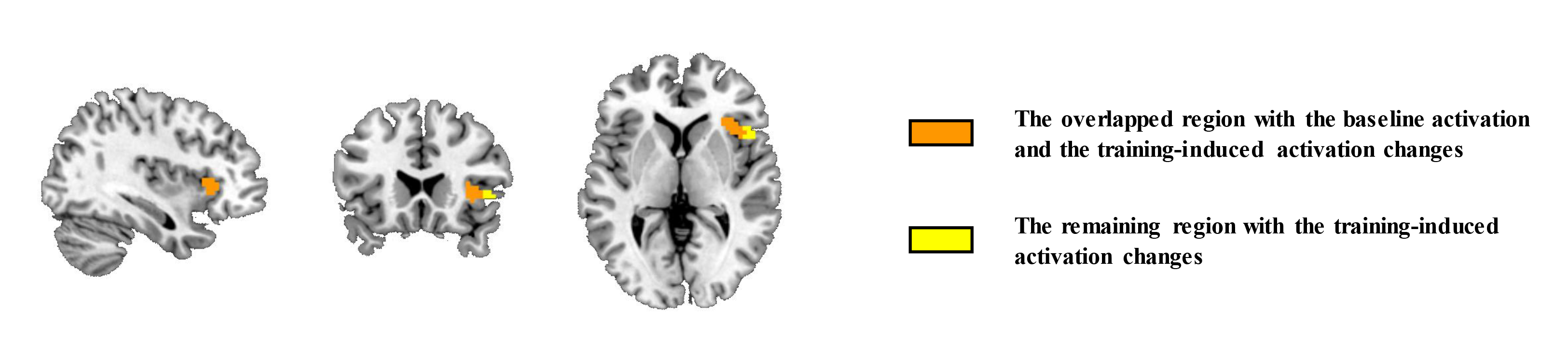

Supplement: Supplementary file 1 [file S0033291720000720sup.zip › S0033291720000720sup002.tif]
